# Remote learning and mental health during the societal lockdown: a study of primary school students and parents in times of COVID-19

**DOI:** 10.1186/s12889-023-16040-9

**Published:** 2023-06-07

**Authors:** Frank Tian-Fang Ye, Xiaozi Gao, Kuen-Fung Sin, Lan Yang

**Affiliations:** 1 Department of Applied Social Sciences, The Hong Kong Polytechnic University, Hong Kong SAR, P. R. China; 2 Department of Early Childhood Education, The Education University of Hong Kong, Hong Kong SAR, P. R. China; 3 Centre for Special Educational Needs and Inclusive Education, The Education University of Hong Kong, Hong Kong SAR, P. R. China; 4 Department of Curriculum and Instruction, The Education University of Hong Kong, Hong Kong SAR, P. R. China

**Keywords:** Primary school, Remote learning, Emotions, COVID-19, Mental health

## Abstract

**Background:**

The COVID-19 pandemic has brought challenges to families around the world. The prolonged school closures in Hong Kong have forced young students to stay at home and adapt to remote learning for over a year, putting their mental health conditions at risk. Focusing on primary school students and their parents, the main objective of our research is to investigate the socioemotional factors and their associations with mental health conditions.

**Methods:**

A total of 700 Hong Kong primary schoolers (mean age = 8.2) reported their emotional experiences, loneliness, and academic self-concept via a user-friendly online survey; 537 parents reported depression and anxiety, perceived child depression and anxiety, and social support. Responses from students and parents were paired to account for the family context. Structural Equation Modeling was used for correlations and regressions.

**Results:**

The results of students’ responses showed that positive emotional experiences were negatively associated with loneliness and positively related to academic self-concept among students. Furthermore, the paired sample results showed that, during the one-year societal lockdown and remote learning period, the socioemotional factors were associated with mental health conditions among primary school students and their parents. Among our family sample in Hong Kong, evidence supports the unique negative association between students-reported positive emotional experiences and parents-reported child depression and anxiety, as well as between social support and parents’ depression and anxiety.

**Conclusions:**

These findings highlighted the associations between socioemotional factors and mental health among young primary schoolers during the societal lockdown. We thus call for more attention to the societal lockdown and remote learning context, especially since the social distancing practice could be “the new normal” for our society to handle the future pandemic crisis.

## Background

During the past two years, the COVID-19 pandemic has brought great challenges to countries worldwide. Responding to the unprecedented situation, many countries imposed societal lockdown measures to various degrees [1]. With the school closure, students in kindergartens and primary schools were stuck at home with their parents and had little space or time for themselves. Recent evidence suggests that young students have had learning difficulties and even mental health problems during the lockdown [2–4].

Until February 2022, the spread of COVID-19 was relatively well controlled in Hong Kong without any massive outbreak. However, this also means Hong Kong has been implementing strict social distancing measures since the beginning of the pandemic. When the current study was conducted (August 2021), only a few schools in Hong Kong resumed half-day in-person sessions, while others remained closed and kept running remote lessons [5]. While young children had been homeschooled for over a year, many families were coping with considerable pressure due to the pandemic risk and financial-related stress, caregiving difficulties, and family relations [6]. Therefore, it is critical to understand the mental health conditions, homeschooling status, and family relations during the lockdown to facilitate developing interventions and resources for families with young children.

### Lockdown, remote learning, and mental health

The prolonged school closures forced the education system to adapt to online teaching mode, and remote learning became a common form of schooling available [7]. However, the unprecedented long-lasting duration of the remote learning mode posed challenges and obstacles to young students. Young students are a vulnerable group who may experience significant mental health challenges due to the COVID-19 pandemic. They are prone to the impact of societal lockdown and remote learning as they have not yet fully developed self-regulated learning abilities and coping skills. According to UNICEF[8], young children could feel the impact of COVID-19 on their mental health and well-being for many years to come, especially those who have been directly affected by lockdowns, school closures, and loss of education. Moreover, according to the report by the American Psychological Association[9], some of the typical indicators teachers may use to identify students experiencing mental health difficulties may not be available during online instruction phases, which can make it harder to provide timely and adequate support for students who may need it. On the other hand, young children’s learning progress is primarily associated with cognition, motivation, and socio-emotional factors [10]. For example, recent studies suggested that remote learning has been challenging for students, with attention difficulties, boredom, technical problems, a lack of community, low motivation, and poor academic performance [4, 10–14].

Remote learning can lead to reduced social interaction compared to face-to-face classes, which may create psychological problems for students. Studies conducted in Australia and China found that students lacked peer engagement and real-time interaction with teachers [15, 16]. The isolated learning environment during societal lockdown was associated with depression, anxiety, stress, and loneliness among adolescents in Denmark and the USA [17, 18]. Loneliness was also reported as a negative experience in remote learning by students in Peru and Finland [19, 20]. Although modern technology has enhanced communication channels, physical separation due to lockdown may still result in negative emotional experiences and mental health problems.

The potential impact of remoting learning on primary schoolers is of concern. On the one hand, academic-related stressors, such as exams, excessive homework, peer pressure, and academic performance, are common risk factors that affect students’ psychological well-being [21–24]. On the other hand, the isolated learning environment may worsen academic performance and put students’ mental health at risk [25, 26]. To date, little attention has been paid to the impact of remote learning on primary schoolers, especially those of young ages (e.g., Primary 1). It is likely because surveying young students were challenging due to their limited reading comprehension abilities. For example, studies focusing on primary schoolers only used parental reports, interviews, and academic performance [3, 4, 10].

### Remote learning and mental health in family context

As the pandemic forced children to learn from home, parents had to take on the role of teachers, which led to increased stress and work-life balance challenges. A study of German and Mexican parents found that they faced difficulties with pedagogical tasks and technical issues while assisting their children with remote learning [3]. Less prepared parents experienced higher depression, parenting stress, and burnout [27, 28]. The pandemic also brought emotional and psychological challenges to families, endangering family relationships and mental health conditions among family members [2, 29–31]. Parenting stress and anxiety were positively associated with child anxiety, while low levels of parent-child communication indicated a higher level of child depression [28, 32]. A poor parent-child relationship also indicated more academic problems among adolescents [33]. However, these findings mainly relied on reports from one family member (i.e., parents or children). Thus, it is necessary to conduct research in the family context to support the development of mental health interventions and the implementation of diverse teaching practices.

### The current study

Considering the arduous nature of societal lockdowns, our research aims to:

- Investigate the mental health status of Hong Kong families, particularly primary school children and their parents;
- Examine the socioemotional factors potentially associated with the mental health conditions of these families;
- Explore the familial impact of these conditions by analyzing the associations between students and their parents.

## Methods

### Participants

The current study was conducted during the COVID-19 pandemic while social distancing and societal lockdowns were still implemented in Hong Kong (from May to August 2021). Before collecting data, we performed a Monte Carlo simulation [34] to determine the optimal sample size. The results suggested that, to identify a 20-item measurement model with moderate factor loadings (lambda = 0.50) that would yield good model fit indices (CFI > 0.95, TLI > 0.95, RMSEA < 0.06, SRMR < 0.06), 380 participants were required. In collaboration with an NGO, the surveys were distributed to 11 Hong Kong primary schools associated with the NGO across all territories in Hong Kong. The schools were instructed to distribute survey invitations to all primary 1–3 students, and the students and their parents were free to participate. Thus, the current study used convenient sampling methods. The surveys for students and parents were collected separately. Following data collection, a planned data cleaning procedure was carried out. Surveys with accidental clicks (no data input), apparent duplicates, or a considerable number of incomplete questions (e.g., 60%) were excluded. Furthermore, data from students and parents were matched using anonymous codes for subsequent analysis. A total of 700 student surveys were collected in classrooms with the assistance of teachers. Furthermore, 537 parents were recruited from the same schools and participated in the survey through invitations sent by the NGO and schools via separate emails and messages.

It is important to note that the students and parents in the current study were not recruited using randomization methods. Due to the half-day teaching arrangement and social distancing measures that were in place during the data collection period, the NGO decided to use a convenient sampling method and collect data from its associated schools. However, the final sample of students had a balanced distribution of gender, age, and grade, while the sample of parents closely matched the SES of the Hong Kong population. The anonymized datasets were obtained with prior agreement from a local NGO. The NGO reviewed the ethics and conducted the data collection. Before filling out the surveys, verbal briefings were given by teachers, and verbal consent was obtained from all students. Parents were given informed consent at the beginning of the online survey, which included information about both their own and their children’s participation. Parents were informed about their children’s involvement and had the option to withdraw their children from the study by notifying the teacher. The information sheet and consent form emphasized that all participants had the right to withdraw from the study at any time without consequences. A total of 761 responses were initially collected from students. However, 61 of these responses contained missing data exceeding 60% and were consequently excluded from the subsequent analyses. To match data collected from students and their parents, pre-defined anonymous family codes were utilized. Out of the successfully matched samples, only seven families had complete data from both parents. Therefore, the data of fathers were removed from subsequent analysis. As a result, the current study retained 249 pairs of mothers and students.

### Measures

#### Student survey

The survey was distributed during the half-day face-to-face class arrangement period, and students were instructed to complete a brief online survey using computers and tablets provided by the school. To accommodate the reading ability among young students, we designed an online questionnaire, which was compatible with tablets and with a user-friendly interface, to facilitate the data collection among primary schools. The survey adopted big fonts, simple choices, and age-appropriate Cantonese for young students to comprehend the questions. In addition, all survey instructions, questions, and options were pre-recorded by a research assistant, and the recordings were placed next to their corresponding survey questions. Hence, students could feasibly click the play button, listen to the questions, and make simple choices. Thus, instead of using parent/teacher reports, the current study collected first-hand self-report survey data among primary school students with limited reading comprehension abilities. Other than demographic questions, the included measures were described below.

**Emotional Experiences**. Three items (joy, anger, bored) were chosen from the Achievement Emotions Questionnaire (AEQ; 35) to assess students’ emotional experiences during the social lockdown. The short form and its Chinese translation were adopted from a previous study of over 8,000 Chinese secondary students [36]. Students were asked to indicate if they had experienced (1 = Agree and 2 = Disagree) these feelings during the past year of remote learning. A sample item included “I felt online classes were boring.“ The item assessing joy was reversed, so the current measure indicated positive emotional experiences. In the current study, McDonald’s ω = 0.67, 95% CI = [0.63, 0.71].

**Loneliness**. Two items with the highest factor loadings and good face validity in previous research were selected from the Revised UCLA Loneliness Scale [37] to measure loneliness. Students were asked to rate whether they felt lonely and whether they felt they had no friends in the past year. Items were rated on a 3-point Likert scale (1 = never, 2 = sometimes, 3 = always). The correlation between the two items was 0.46, 95% CI = [0.40, 0.52].

**Academic Self-concept.** Students’ perceived academic performance was assessed using four items adapted from the Chinese version of Self-Description Questionnaire-I [38], initially developed by Marsh [39]. The four items demonstrated high factor loadings and excellent face validity in previous research [38]. Students were asked to indicate whether they agreed or disagreed with these items. Sample items included “I like most academic subjects.“ In the current study, McDonald’s ω = 0.61, 95% CI = [0.56, 0.65].

**Life Routine During Lockdown.** Students were asked to indicate whether they had enough sleep, outdoor activities and if they had spent time with their peers. They were also asked if they felt their parents were annoying at home during the lockdown.

#### Parent survey

Parents were invited to complete an online questionnaire consisting of the following measures.

**Depression and Anxiety.** The 4-item Patient Health Questionnaire [40] was used to assess parents’ depression and anxiety in the past year. The questionnaire included two items measuring depression (“Little interest or pleasure in doing things”, “Feeling down, depressed, or hopeless”) and two items measuring anxiety (“Feeling nervous, anxious or on edge”, “Not being able to stop or control worrying”). This ultra-brief measure has demonstrated good reliability and validity in previous research [40]. In the current study, parents indicated the frequency of their feelings in the past year on a 4-point Likert scale (1 = less than one day per week, 4 = five to seven days per week). In the current study, McDonald’s ω = 0.89, 95% CI = [0.87, 0.92].

**Child Depression and Anxiety**. The identical items in PHQ-4 were used to assess parents’ perception of their child in the past year. In the current study, McDonald’s ω = 0.81, 95% CI = [0.76, 0.85].

**Social Support**. Parents were asked to indicate to what extent they had received social support during the lockdown. Three items adapted from Zimet et al. [41] were rated on a 3-point Likert scale (1 = never, 2 = sometimes, 3 = frequently). In the current study, McDonald’s ω = 0.67, 95% CI = [0.62, 0.72].

### Statistical analysis

Descriptive statistics of the measures mentioned above were calculated using jamovi [42]. The correlations of the measures were explored using Structural Equation Modeling (SEM) to take the measurement errors into account. Three correlational SEMs were conducted separately for students, parents, and parent-child pairs to maximize the sample size available in each model. In the present study, our SEMs were specified and tested explicitly in accordance with our research questions, and these models are specified as simply as possible to follow the parsimony principle. To actively and effectively account for the measurement error, we used the correlations extracted from SEM rather than conventional Pearson bivariate correlations with composite scores. All items in the measurement model were treated as categorical, and all SEMs were estimated with mean and variance adjusted weighted least squares (WLSMV) estimator using lavaan [43] in R. Models were compared based on the model χ^2^ test statistics, the Comparative Fit Index (CFI), the Tucker-Lewis index (TLI), the root mean square error of approximation (RMSEA), the standardized root mean square residual (SRMR). Because the χ^2^ significance test was sensitive to large sample sizes, it was not taken into account when choosing the best model. To evaluate the model fit, CFI and TLI ≥ 0.90, RMSEA and SRMR ≤ 0 0.08 represented an acceptable model fit, and CFI and TLI ≥ 0.95, RMSEA and SRMR ≤ 0.06 represented a good model fit [44, 45].

The dataset and model output files are available on the OSF page via an anonymized link (https://osf.io/2a9pr/?view_only=05fc816fc07042d99e69eef8186ba1c7).

## Results

For the students sample (N = 700), 51.4% were females, the mean age is 8.2 (SD = 1.06) and ranged from 5 to 13 (Primary 1 to 3. For the parents sample, similar to other studies in the field, the majority (84.1%) of the caregivers in the sample were mothers. The other demographic information of parents can be found in Table 1.

**Table 1:** *Demographic Information Reported by Parents*

	Frequency	Percentage		Frequency	Percentage
Parental Role			Monthly Household Income		
Father	85	15.9%	<HKD10000	46	8.6%
Mother	451	84.1%	HKD10001 - HKD20000	91	17.1%
**Child Gender** (Enrolled in the surveyed schools)			HKD20001 - HKD30000	78	14.7%
Male	280	52.30%	HKD30001 - HKD40000	62	11.7%
Female	255	47.70%	HKD40001 - HKD50000	49	9.2%
**Number of Kids at Home**			HKD50001 - HKD60000	45	8.5%
1	189	35.2%	HKD60001 - HKD70000	30	5.6%
2	292	54.4%	HKD70001 - HKD80000	28	5.3%
3	44	8.2%	HKD80001 - HKD90000	21	3.9%
4	11	2.0%	HKD90001 - HKD100000	21	3.9%
5	1	0.2%	HKD100001-HKD110000	21	3.9%
**Education**			HKD110001 - HKD120000	8	1.5%
Lower than High School	15	2.8%	HKD120001 - HKD130000	5	0.9%
High School	209	38.9%	HKD130001 - HKD140000	5	0.9%
Associate Degree	108	20.1%	HKD140001 - HKD150000	3	0.6%
Bachelor’s Degree	153	28.5%	>HKD150001	19	3.6%
Postdoctoral Degree	52	9.7%			

*Note*. The median overall monthly household income in Hong Kong was 34,500 HKD in 2020 [46]. Some participants did not provide all demographic information

Descriptive statistics of measured variables were presented in Table 2.

**Table 2:** *Descriptive Statistics of Study Survey*

Item	Answer (N)	Missing(N)
Outdoor Activities	Decreased: 410 (58.8%); Same as before:192 (27.5%); Increased: 95 (13.6%)	3
Sleep	Not enough: 218 (31.4%); Enough: 476 (68.6%)	6
Annoying Parents	Never: 154 (22%); A little: 408 (58.3%); Very much: 138 (19.7%)	0
Time with Peers	Never: 433 (61.9%); Sometimes: 210 (30%); Frequently: 57 (8.1%)	0
Joy	Agree: 306 (43.8%); Disagree: 393 (56.2%)	1
Anger	Agree: 389 (55.8%); Disagree: 308 (44.2%)	3
Bored	Agree: 340 (48.9%); Disagree: 356 (51.1%)	4
Loneliness 1	Never: 380 (54.5%); Sometimes: 201 (28.8%); Always: 116 (16.6%)	3
Loneliness 2	Never: 368 (52.7%); Sometimes: 236 (33.8%); Always: 94(13.5%)	2
Academic Self-concept 1	Agree: 331 (47.4%); Disagree: 367 (52.6%)	2
Academic Self-concept 2	Agree: 264 (37.9%); Disagree: 432 (62.1%)	4
Academic Self-concept 3	Agree: 443 (63.7%); Disagree: 252 (36.3%)	5
Academic Self-concept 4	Agree: 407 (58.5%); Disagree: 289 (41.5%)	4

The datasets for both parents and students were assessed using Little’s MCAR test in R to examine the missing data. The results indicated that the parents’ data was missing completely at random (p = .58), while the students’ data was missing at random (< 0.9% missing with p = .01, no significant correlations between missing indicators and non-missing indicators).

For the correlational analyses, the model estimating the student sample yielded an acceptable model fit (Robust χ^2^ = 186.996, df = 54, CFI = 0.931, TLI = 0.900, RMSEA = 0.061, 95% CI [0.051, 0.070], SRMR = 0.072). Standardized factor loadings ranged from 0.341 to 0.926, and were significant at *p* < .001 level. The results showed that positive emotional experiences were positively associated with academic self-concept, and negatively associated with loneliness; academic self-concept was also negatively associated with loneliness (see Table 3 for details). These findings supported the notion that social emotional factors were associated with students’ academic functioning. Additionally, positive emotional experiences was positively associated with outdoor activities, β = 0.24, p < .001, 95% CI [0.14, 0.35], and was negatively associated with annoying parents, β = -0.14, p < .01, 95% CI [-0.25, -0.04]; surprisingly, it was not related to time with peers, β = 0.001, p = .98, 95% CI [-0.11, 0.11], or sleep quality, β = 0.11, p = .07, 95% CI [-0.01, 0.24]. Loneliness was positively associated to annoying parents, β = 0.14, p < .01, 95% CI [0.04, 0.24], and was negatively associated with time with peers, β = -0.12, p < .05, 95% CI [-0.23, -0.01], and sleep quality, β = -0.17, p < .01, 95% CI [-0.29, -0.05]; however, it was not associated with outdoor activities, β = -0.07, p = .20, 95% CI [-0.17, 0.04]. Lastly, academic self-concept was positively correlated to time with peers, β = 0.15, p < .01, 95% CI [0.05, 0.26], sleep quality, β = 0.19, p < .01, 95% CI [0.07, 0.30], and outdoor activities, β = 0.18, p < .01, 95% CI [0.08, 0.28]; it was negatively associated with annoying parents, β = -0.20, p < .001, 95% CI [-0.30, -0.10]. These findings also suggested potential impact of social environment on students’ daily functioning during the society lockdown.

The model estimating the parent sample yielded a good model fit (Robust χ^2^ = 65.463, df = 41, CFI = 0.996, TLI = 0.995, RMSEA = 0.035, 95% CI [0.018, 0.050], SRMR = 0.041). All factor loadings were significant at p < .001 level and ranged from 0.611 to 0.941. The results showed a positive correlation between parents’ reported depression and anxiety and perceived child depression and anxiety, as well as negative correlations between social support and depression and anxiety of both parties (see Table 3). The results demonstrated consistent parental perception, indicating that parents’ self-rated mental health conditions were in line with their assessment of their children’s conditions. Additionally, seeking social support during the lockdown could potentially benefit both parties.

**Table 3:** *Latent Correlations of Measurements for Students and Parents*

	1	2	3	4	5
Students					
1 Emotional Experiences (Positive)	-				
2 Loneliness	-0.30*** [-0.42, -0.18]	-			
3 Academic Self-Concept	0.27*** [0.15, 0.38]	-0.22*** [-0.33, -0.10]	-		
**Parents**					
4 Depression and Anxiety	-0.09 [-0.29, 0.11]	0.07 [-0.13, 0.26]	-0.01 [-0.18, 0.16]	-	
5 Child Depression and Anxiety	-0.32** [-0.52, -0.11]	0.26** [0.07, 0.45]	0.05 [-0.13, 0.23]	0.63*** [0.55, 0.71]	-
6 Social Support	0.13 [-0.08, 0.33]	-0.17 [-0.39, 0.04]	0.27** [0.07, 0.47]	-0.18** [-0.31, -0.06]	-0.18** [-0.30, -0.05]

Note. The above bivariate latent correlations were extracted from three separate SEMs were conducted for students (*N* = 700), parents (*N* = 537), and parent-child pairs (*N* = 249)

** p < .01, *** p < .001

Similarly, the model estimating the paired sample also yielded a good model fit (Robust χ^2^ = 173.010, df = 155, CFI = 0.994, TLI = 0.993, RMSEA = 0.024, 95% CI [0, 0.042], SRMR = 0.076). Standardized factor loadings ranged from 0.511 to 0.985, and were significant at *p* < .001 level. Perceived child depression and anxiety were negatively correlated to students’ positive emotional experiences and positively correlated to their loneliness, suggesting a consistent self-perception of mental health conditions between students and their parents. A significant positive correlation was observed between students’ academic self-concept and the social support reported by their parents, as presented in Table 3. However, such a correlation was not found for the students’ emotional experience or feelings of loneliness. This may indicate that parents primarily seek social support for their children’s academic tutoring.

**Fig. 1:**
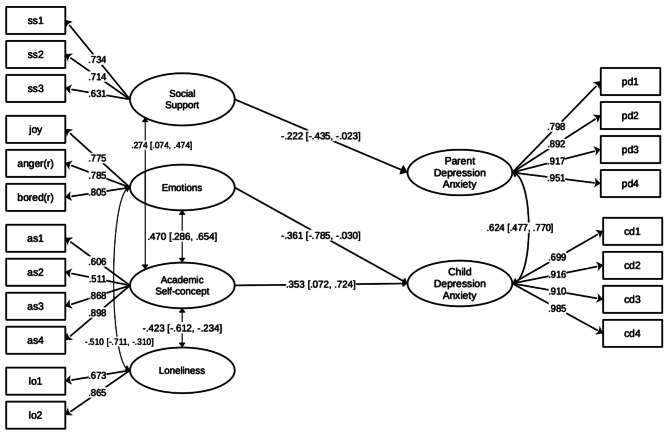
Structural Equation Model demonstrates the associations in the family context *Notes*. Anger and bored were reversely coded. Standardized factor loadings, path coefficients, and covariances were reported in the figure. The 95% confidence intervals were included in the brackets. Non-significant paths and covariances were omitted for clarity

Given the nature of the cross-sectional design, we refrained from making causal inferences in the current study. However, an additional regression model was tested using SEM to explore the unique associations of these variables, in which mental health variables were regressed on emotional experiences, loneliness, academic self-concept, and social support. The regression model yielded an identical model fit index to the correlational model, as no additional variables were included. After controlling for the covariates, the results showed that social support was the only significant predictor of parents’ depression and anxiety (β = − 0.222, p = .029, 95%CI [-0.435, − 0.023]), and emotional experiences and academic self-concept were significant predictors of child depression and anxiety (β = − 0.361, p = .034, 95%CI [-0.785, − 0.030] and β = 0.353, p = .017, 95%CI [0.072, 0.724]), suggesting unique impacts of these three factors on the mental health conditions among family members. The model was visualized in Fig. 1.

## Discussion

The current study surveyed primary school students and their parents in Hong Kong during the societal lockdown in 2021, and investigated the socioemotional factors and their associations with mental health conditions. The results of the students’ responses revealed a negative correlation between positive emotional experiences and loneliness, as well as academic self-concept. Furthermore, after controlling for covariates, regression analyses using structural equation modeling demonstrated that socioemotional factors had unique impacts on the mental health of primary school students and their parents during this period.

The study has the potential to make several contributions to the existing literature. Firstly, it used young children’s self-reports to gain insights into their internal experiences and mental health, which is a viable means of understanding primary schoolers’ mental health conditions. Previous research has seldom focused on mental health conditions among young primary school students, primarily due to the difficulty of implementing self-report surveys for students with limited reading comprehension abilities. Our study, however, took advantage of technological advancements in Hong Kong during the COVID-19 pandemic and designed a user-friendly online survey suitable for young children, offering essential insights into the first-person experience of eight-year-old students and their psychological conditions. Secondly, by connecting the student and parent samples, our study captured the family context and tested the associations of mental health between family members. As both parties responded to the survey independently, the results provided a relatively objective view of family relations. Lastly, our study focused on a vulnerable group in response to the call for attention to the post-pandemic mental health crisis. The group, facing academic pressure and transitioning from daycare elementary school to an independent self-learning environment, was further challenged by the one-year-long lockdown, no face-to-face peer interaction but only virtual classrooms.

As expected, the findings of the student sample showed that positive emotional experiences were associated with lower levels of loneliness and better academic self-concept, while loneliness was negatively associated with academic self-concept. Furthermore, correlational analyses also revealed that spending time with peers, engaging in outdoor activities, and good sleep quality were significant contributors to a healthy mental state. Linking the student sample to the parent sample further revealed a negative association between positive emotional experiences and child depression and anxiety, as well as a positive association between loneliness and child depression and anxiety. These findings suggest that students’ emotional experiences played a crucial role in their self-perception of academic performance and mental health during the homeschooling and remote learning process. To promote the mental health and well-being of primary school students under such circumstances, parents can encourage outdoor activities, ensure adequate sleep, and provide opportunities for peer interaction; teachers can incorporate socioemotional learning programs, promote physical activity, and be attentive to signs of distress; policymakers can increase funding for mental health services, implement socioemotional learning programs in the national education curriculum, encourage physical activity, and provide guidelines for healthy screen time habits.

In the current study, the parents’ report showed that social support was not related to students’ mental health, emotional experiences, or loneliness but only to academic self-concept. This phenomenon may partially be a manifestation of the typical culture in Hong Kong, where parents primarily seek help for kids with their homework (i.e., private tutoring) rather than psychological needs [47]. Hence, promoting mental health-related knowledge among parents and providing widely accessible mental health support to primary school students should be emphasized in our society.

Attention must be paid to the isolated learning environment for kids, especially under the societal lockdown that a systematically isolated norm was implemented. Moreover, remote learning settings, such as virtual classrooms and online courses, have been susceptible to the criticism that they lack emotional exchanges and peer interactions and commonly adopt one-way teaching [48], which may not facilitate effective learning but induce more mental health problems as mentioned above. Therefore, psychological support in classrooms is needed under such circumstances. We suggest that parents and teachers get involved in children’s learning process by coaching, initiating conversations, and providing emotional support when children become frustrated. Most importantly, schools, teachers, and parents need to innovatively adapt online courses into a format with more fun activities, parent-child interactions, and peer collaborations to minimize and counteract boredom and loneliness. Educators and parents can consider organizing virtual peer-to-peer activities, encouraging children to participate in group discussions, providing opportunities for collaborative projects, and offering counseling services for mental health support. It is essential to find a balance between academic progress and emotional well-being in the isolated learning environment.

Furthermore, it is noted that students’ academic self-concept demonstrated a positive association with their depression and anxiety after controlling for other covariates. This finding indicates that perceived academic performance partially explained the variance of depression and anxiety beyond emotions, loneliness, and social support, suggesting that the aversive impact of academic stress on mental health could be masked by socioemotional factors. Promoting positive emotions, reducing loneliness, and even seeking social support with logistics and homework may be helpful to students’ academic performance, but it could also indirectly induce more mental health problems. These results corroborate existing literature that highlights the influential role of academic stress in young children’s school life as it might serve as a double-edged sword [49] and encourage educators to pay attention to young students’ mental states associated with their academic performance.

Particularly notable is that nearly 60% of the students in the current study reported their parents being “a little bit annoying” during the lockdown, and around 20% of them rated their parents “very much annoying.“ Moreover, the correlational analyses revealed that students who felt more strongly annoyed by their parents tended to report having lower positive emotions, stronger loneliness, and weaker academic self-concept. The findings suggest that parents should be mindful of their behavior and communication with their children during the lockdown to ensure positive family relations. Parents can make an effort to create a supportive and positive atmosphere at home by engaging in fun activities with their children, being empathetic and understanding of their emotions, and creating a schedule that balances schoolwork and leisure time. Communication is key, and parents should encourage open dialogue and actively listen to their children’s concerns. Educators can also play a role in supporting families by offering guidance and resources to improve family communication and relationships. Educators and caregivers should prioritize the mental health of young students during these challenging times, as it can impact their academic performance and overall well-being. Furthermore, policymakers can also promote family-friendly policies, such as flexible work schedules, paid leave, and affordable childcare, to reduce stress and anxiety for parents and allow them to spend more time with their children during the difficult times.

The overall results from the current study call for more attention to remote learning context, especially since the social distancing practice could be “the new normal” for our society to handle the future pandemic crisis. The impact of such isolation may be even more significant in the pandemic era as the lockdown measures were enforced by the government and schools, affecting a broad range of students in society. Moreover, the impact of such a global norm could induce long-term changes in child and adolescent socioemotional development [14].

All the implications raised from the current study suggest that young students’ mental health conditions during societal lockdown are not solely the responsibility of parents. It is also closely associated with educators and decision-makers. In this light, remoting learning and academic achievement are not the solutions or only needs for young students; emotional needs, peer relations, and parent-child relations are crucial factors and should be emphasized in societal lockdowns.

This study is not without limitations. First, due to the nature of the cross-sectional design, we could not draw causal inferences from the data. Future research should conduct longitudinal studies to study the dynamic influence of risk factors on young children’s mental health. Second, to accommodate primary schoolers’ limited reading comprehension ability, the self-report survey only contained limited measures. Although the students’ personal experiences were directly assessed, we acknowledge that the short measurements might not cover the whole story. Therefore, qualitative research and multitrait-multimethod designs [50] could be conducted in future research to examine other factors associated with mental health in the societal lockdown and remote learning context. Furthermore, we utilized modified short measurement tools for the student survey for similar reasons, which could potentially affect the validity of the measured concepts. Therefore, caution is advised when interpreting the findings of this study.

## Conclusion

The current study found a negative correlation between positive emotional experiences and loneliness, as well as a positive correlation between positive emotional experiences and academic self-concept, based on students’ responses. The paired family sample results revealed that socioemotional factors were linked to mental health issues among primary school students and their parents during the one-year period of societal lockdown and remote learning. The results of the current study provided further evidence supporting the unique negative association between students’ reported positive emotional experiences and parents’ reported child depression and anxiety, as well as between social support and parents’ depression and anxiety after controlling for various covariates. We thus call for more attention to the societal lockdown and remote learning context, especially since the social distancing practice could be “the new normal” for our society to handle the future pandemic crisis.

## Data Availability

The anonymized datasets and results discussed in the manuscript were uploaded on OSF and shared anonymously (https://osf.io/2a9pr/?view_only=05fc816fc07042d99e69eef8186ba1c7).
